# Precision Medicine: Making It Happen for Malaysia

**DOI:** 10.21315/mjms2021.28.3.1

**Published:** 2021-06-30

**Authors:** A Rahman A Jamal

**Affiliations:** Paediatric Haematology, Oncology and Molecular Biology, Universiti Kebangsaan Malaysia, Kuala Lumpur, Malaysia

**Keywords:** precision medicine, cancers, rare diseases, non-communicable diseases, genome, electronic medical record, better outcome, value

## Abstract

Precision medicine is transforming healthcare worldwide and aims to improve the effectiveness of management of many diseases including cancers, other non-communicable diseases (NCDs) and also rare diseases. Precision medicine takes into account the individual patient’s genetic, environment and lifestyle data. Developed nations are already embarking on precision medicine initiatives including the 100,000 Genomes England and the Precision Medicine Initiative in the United States (US). The Academy of Sciences Malaysia, the Ministry of Health and the Ministry of Higher Education are working together to put forward a precision medicine initiative for Malaysia. The key drivers that must be put in place include a strong policy agenda, a national large scale genome sequencing project and with it a national genome database, the implementation of the electronic medical record (EMR) system, a payment and reimbursement system to cover for the genetic testing and the targeted treatment, and putting in place an ecosystem that will support precision medicine. Relevant guidelines and Acts will also need to be developed especially with regard to privacy and confidentiality. The future of precision medicine is now and this will certainly bring better outcome and value to the patients.

**Figure:**
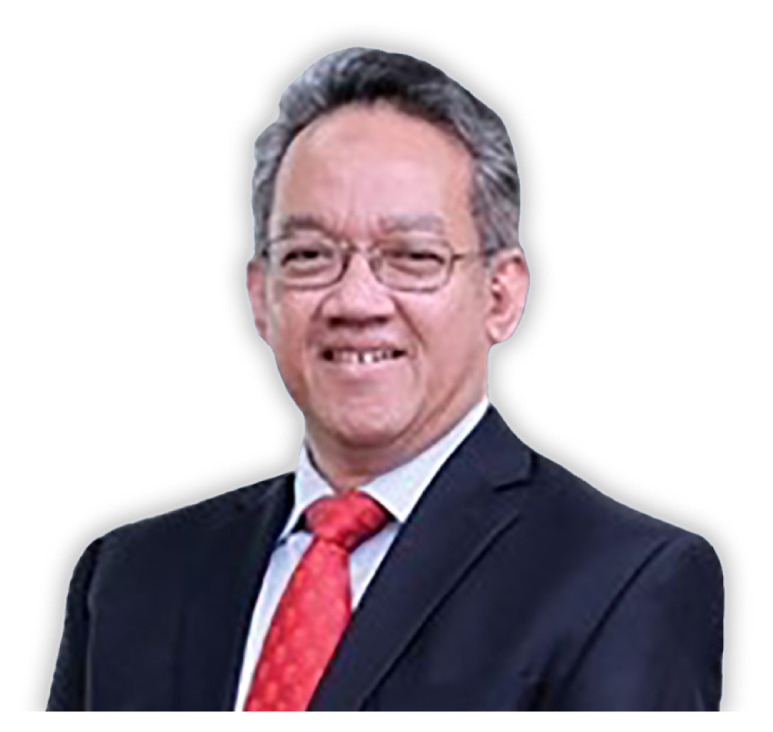


Precision medicine has now become a key initiative to transform healthcare in many developed and developing nations. What started as personalised medicine, it has evolved into a more encompassing concept of healthcare that takes into account the individual uniqueness in terms of genetic, environmental exposure and lifestyle data. The expanded definition of precision medicine is the tailoring of medical treatment to the individual characteristics of each patient aiming to classify individuals into subpopulations that differ in their susceptibility to a particular disease or their response to a specific treatment (1). The targeted outcomes include better diagnosis, improved decision-making processes, more effective treatment, reduced wastage through avoiding unnecessary diagnostic testing and therapies, and also better disease prevention strategies.

The world has seen how the United Kingdom (UK) launched its precision medicine initiative in a big manner in 2012 with the 100,000 Genomes England project that involves genome sequencing of cancers, pathogens and rare diseases (2). The project was so successful that the UK government agreed to extend and expand the programme to sequence 5,000,000 individuals. The United States (US) soon followed suit with the announcement, by President Barrack Obama himself, of its own Precision Medicine Initiative in 2015 (3). One of the core programmes in the initiative is a cohort of 1,000,000 individuals, named ‘All of US’ that will have participants contribute their healthcare data as well as their genome data to a central database. In addition to these two big initiatives, many other countries have embarked on similar programmes albeit on a smaller scale. The precision medicine initiative indeed has arrived in a big manner, hence it is strategic that Malaysia begin to plan on how to use this to transform our healthcare. Indeed this will be a major paradigm shift especially so for countries with limited resources, but change is inevitable especially it is for the better.

Precision medicine is already being practised locally especially in the field of oncology. The use of cetuximab, which is anti-epidermal growth factor receptor (EGFR), in colorectal cancer is guided by the presence of Kirsten rat sarcoma viral oncogene homolog (KRAS) gene mutations in the tumours. KRAS mutations are a predictor of resistance of cetuximab (4). Another application is in non-small cell lung cancer in which the presence of EGFR mutations identifies patients who will benefit from treatment with EGFR tyrosine kinase inhibitors (5). It is interesting to note that 32% of Asian patients with non-small cell lung cancer (NSCLC) have EGFR mutations compared to just 7% of patients of other ethnic groups. The third example in oncology is the use of the Oncotype Dx testing for breast cancer which determines the likelihood of recurrence of the cancer and whether the patient is likely to benefit from chemotherapy in early stage invasive cancer type (6). The three cancers cited here are in the top five cancers in Malaysia as well as globally. Unfortunately, these tests are not widely available and many patients will have to pay out of their pockets to get tested. It is clear that implementing precision medicine will increase the cost (due to the testing), nevertheless the outcomes of treatment will be much improved. In health economics, this constitutes the value of knowing and the value in terms of making the best treatment decision for each patient.

There are also other applications of precision medicine, using genetic testing, in the local context as well. A good number of laboratories are already offering the test for the HLA-B*1502 allele which is highly associated with Steven-Johnson syndrome and toxic epidermal necrolysis, in epilepsy patients treated with carbamazepine (7). Most pharmaceutical regulatory authorities have made it mandatory to have this test before prescribing carbamazepine to epilepsy patients. This allele is more common in Asians compared to Caucasians. Certain cancers have a heritability component including breast and colorectal cancer. This is where precision medicine is applied to identify individuals of risk of getting these cancers and hence allowing closer monitoring, early detection as well as preventive mastectomy and colectomy. For breast cancer, the genes involved are the breast cancer gene 1 (BRCA1) and breast cancer gene 2 (BRCA2) genes whilst mutations in the adenomatous polyposis coli (APC) gene, which plays a key role in sporadic colorectal cancers, is also responsible for the familial adenomatous polyposis. In addition, those who have Lynch syndrome also known as the hereditary non-polyposis colorectal cancer, has increased risk of getting colorectal cancer (and other cancers too), and at a younger age. Those with a strong history of these cancers in their families are encouraged to have genetic testing done. Another impactful application of precision medicine is in the management of rare diseases. It is estimated that one in 17 people in the UK suffers from a rare disease. Rare diseases when taken together are actually not rare. Many patients with rare diseases are subjected to so many investigations before a diagnosis is made and in many of them the diagnosis remain unknown. The use of whole genome or whole exome sequencing has been shown to achieve a diagnosis in up to 40% of the cases and may even identify a defect in a particular metabolic pathway that could be treated.

What are the five key initiatives that must be in place to make the precision medicine initiative successful in Malaysia? First and foremost, a policy agenda should be put in place and a clear strategic plan be implemented. It is assuring to know that the Academy of Sciences Malaysia (ASM) has recently put forward a position paper on the Precision Medicine Initiative for the nation (8). This was followed by a joint paper between ASM and the Ministry of Health on precision medicine, digital health and clinical trial hub, which was presented to the National Science Council chaired by the Prime Minister. The second initiative is to roll out a national genome sequencing initiative to test and profile patients with different diseases (cancers, diabetes, heart disease, rare diseases and also those with COVID-19 infections). The genome data from this initiative, even if we start with just 10,000 genomes, will be valuable as it will give us crucial information in terms of key genetic variants involved in many different diseases as well as identifying targeted therapies for those with cancers and informing those at risk of diseases so that they could be put on effective preventive programmes. It is crucial that we continue to generate evidence that precision medicine is cost effective and improves the outcome and quality adjusted life years in our patients. The genome data from our nation will also be filling the knowledge gap as current genome databases globally lacks data from this part of the world. The third key thing to do is much related to the information and communication technology (ICT) as well as the big data component. The electronic medical record (EMR) system should be implemented throughout all hospitals. Data from the EMR coupled with gene sequencing can identify the gene variants and mutations associated with disease and the different phenotypes. We also need to develop the capacity and capability in data science, data analytics and artificial intelligence, as once the precision medicine initiative are in place, large scale datasets will be collected and stored. Issues on ICT infrastructure, interoperability, harmonisation of data standards, and human resource must also be addressed simultaneously. The fourth component will be related to payment and reimbursement. To this end, arguably the best approach will be a social health insurance scheme that will subsidise or pay for the genetic testing as well as the treatment that is coupled to the test especially for those with cancers and rare diseases. If the precision medicine initiative is to be successful, affordable and accessible by everyone, there must be a commitment to implement a sustainable funding system. The last key initiative to do is to ensure that the components in the precision medicine ecosystem will work together. There must be buy-in from the public, patients and clinicians, and this can be achieve via comprehensive education and awareness programmes. Relevant guidelines, regulations and Acts should be developed and put in place to protect privacy and confidentiality of personal data including genetic data. A key Act to be enacted is that related to genetic information non-discrimination to ensure those individuals that have been identified to have variants related to diseases are not deprived of their rights to medical insurance and employment. Laboratories doing the genomics testing need to have the proper accreditation and also allow data sharing so that we have the right volume of collective data to really exploit and make the key discoveries to improve patients’ outcomes. The quadruple helix approach of collaboration that includes the government, academia, industry and the civil society is needed for the precision medicine ecosystem. The role of the industry cannot be understated as this initiative will involve many different industries related to life sciences, pharmaceuticals, ICT, big data, artificial intelligence and also biomedical engineering.

In summary, precision medicine is a major paradigm shift that will transform our healthcare in a big manner and it is assuring to know that the government will be giving full support for the initiative. There are key programmes and activities to be launched to ensure a successful implementation including putting a clear policy agenda and roadmap, a large scale genome sequencing initiative, upgrading the ICT infrastructure and the comprehensive adaptation of the EMR, developing the relevant guidelines and Acts especially relating to privacy and confidentiality, and bringing in together the major stakeholders together to ensure we have the effective ecosystem for precision medicine. The future of precision medicine is now, hence we need to work together to make this transformation in healthcare that will bring better outcome and value for the individual patient, the health of the community and the nation’s economy.
